# Our Bureau of Information

**Published:** 1912-02-03

**Authors:** 


### OUR BUREAU OF INFORMATION.

### A Missing Link in the Hospital Chain.

A short time ago a query was sent to the Editor of The Hospital: "Is there any hospital for incurable patients between the ages of sixteen and twenty-five?" The answer to the question, so far as London is concerned, is practically a negative. There are hospitals for incurable children, where the inmates are kept until they are sixteen years of age, and there are hospitals for adult incurables—*i.e.*, persons over thirty, and in one large institution over thirty-five; but there are only a few beds available for young persons afflicted with incurable disease. One small hospital for women receives patients at any age, and has one inmate who entered when she was seventeen and has been there twenty-five years. Another home for women also makes no restriction; but this is a place where the patients or their friends contribute about three-fourths of the cost of their maintenance. The secretary of this institution writes that there is great need for a similar home for men. In two of the largest institutions admission to the hospital, or election to a pension of £20—which is given to those who have friends with whom they can live—is obtained only through the votes of subscribers. This means in many instances that those who have the largest number of friends are successful; not necessarily the most deserving cases. In the case of one of these institutions it is explicitly stated that no applicant will be received or helped who has had Poor Law relief. This limitation has a certain reasonableness, since the inmates of such a hospital must be prepared to pass the remainder of their lives together, and it is well that the rougher members of society should not be thrust upon those whose bringing-up has been different. We should be glad to know if there is any hospital, in London or elsewhere, where patients are received without regard to age or social status.

On another point also we should be glad of information. What is done for the education of incurable children or any who must, owing to the nature of their disease, pass a long time in hospital? In Copenhagen there is a cripples' home where wonderfully good work—equal to that of any ordinary man or woman—is done by boys and girls, men and women, who are twisted in various ways or are lacking in the requisite complement of legs and arms. They do spinning, weaving, sewing, embroidery; they do wood-carving, carpentry, and upholstering—all by the help of ingenious contrivances which make up for Nature's defects. If children were trained from their youth up, they might be able to do that which would lift them to a considerable extent above dependence. We should be glad to know what is being done.

By means of wise teaching and guidance, given when young, those who are obviously doomed to the lifelong limitations of incurable disease might be trained to achieve some degree of that usefulness and independence which makes for self-respect and happiness. Will any who have done this inform us of their methods and what they have achieved?

### NEEDS AND HELPERS.

### Work for a Middle-aged Man.

"Buckstone" writes: Can you help me to obtain employment for a man of 65, married, a retired schoolmaster with a pension of £30 a year? He has excellent testimonials, is willing to accept a very small salary, and says he would refuse no respectable employment.

This query lies, strictly speaking, outside our sphere, but in some of the infirmaries and also in hospitals for children, teachers are employed to give instruction to child patients. This work is only moderately paid, but it is one for which an elderly man would be suitable.

### Home Wanted for an Epileptic Lady.

"M. W." writes: Can you assist me in finding a home for an epileptic lady, aged about 48, at from 10s. to 12s. weekly? I have tried for years to find an institution suitable, but without success. At present she resides in private families, but constantly has to leave, and should be under proper care, if only it were possible to find a place at the low price named.

(This case might be suitable for the colony belonging to the National Society for Epileptics at Chalfont St. Peter's, Bucks. The charge is 10s. per week, but applicants must be selected by the committee. The Secretary is Mr. G. Penn Gaskell, and the London office is at Denison House, Vauxhall Bridge Road, Westminster, S.W.

### Our List of Approved Homes.

We are pleased to add to our list of approved homes the following: "Cotswold," Ilfracombe. Principal, Miss Catherine Layton. Small Home for invalids and chronic patients. Mild climate.

### Answers and Correspondents.

"K. U." Ipswich.—We do not recommend practitioners.

"Regina."—Unless coupon is sent we cannot insert your query. You will find it at the bottom of the third page of the cover of The Hospital.

"F. W. G., Gosport," writes: Can you furnish me with any information of where I should be likely to obtain a situation in a laboratory of an hospital where I could further increase my knowledge of laboratory work? I may mention that salary would be optional.

"E. E. C." writes: Could you give me addresses of Hospitals in Switzerland where English probationers are taken?

"F. A. H." writes: As I have several hours every week to spare, I should be glad to know if there is any hospital in the London district where a visitor would be welcome, either within the walls or in connection with the work outside.

### A Correction.

We regret that in a recent issue we spoke of the Hospital for Women and Children, 7 Lupus Street. We are now informed that this hospital has recently been closed. We thank our informant for the correction.

